# Supplementary material for: Perceptions of tourists of the resources, ecological service functions and recreation value of the Guanwu National Forest Recreation Area
Source: PLoS One. 2021 Sep 30;16(9):e0257835. doi: 10.1371/journal.pone.0257835 (PMC8483363; doi:10.1371/journal.pone.0257835)
Supplement: S1 File — (DOCX) [file pone.0257835.s001.docx]

親愛的受訪者，您好：

首先，非常感謝您百忙之中撥空填寫本問卷。這是林務局委託國立屏東科技大學森林系執行的研究計畫。而本問卷發放之主要目的，在瞭解您對**「觀霧」**國家森林遊樂區功能價值與發展方向的看法，以俾提供給管理單位作為未來擬定及修正相關政策之參考。

本問卷絕不公開個人資料，敬請安心填寫。而所得調查結果將供學術研究使用，同時也會提供給政府相關單位作為擬定森林遊樂區經營管理策略之參考，因此您的意見十分寶貴。

最後，很感謝您配合撥空回答問題，您的熱誠參與，將是政府單位施政的最佳基石。謝謝！

耑此 祝您

平安

國立屏東科技大學森林系 陳建璋 助理教授 暨研究團隊 敬上

**第一部分 旅遊行為與保育認知**

A1. 請問您此次前來本區旅遊的**同遊夥伴**是誰？

□自己 □家人 □同學 □朋友 □同事 □團體旅遊 □其他

A2. 請問您此次前來本區旅遊所使用的**交通工具**為何？

□機車 □自用車 □遊覽車 □租賃汽、機車 □其他

A3. 請問您此次前來本區旅遊的**交通時間**大約多久？

□1小時 □2小時 □3小時 □4小時 □5小時 □其他

A4. 請問您此次前來本區旅遊大約會**停留多少時間**？

□2小時以內 □2-4小時 □4-6小時（半天） □6-8小時

□8小時以上（一天） □兩天一夜 □三天兩夜 □其他

A5. 請問您對本區自然遊憩資源的評價為何？

□價值非常低 □價值低 □價值普通 □價值高 □價值非常高

A6. 請問您對本區文化遊憩資源的評價為何？

□價值非常低 □價值低 □價值普通 □價值高 □價值非常高

A7. 請問您重遊本區的意願如何？

□非常低 □低 □普通 □高 □非常高

A8. 您是否知道森林遊樂區的供給功能？（如食物、飲用水、林木生產等）□是；□否

A9. 您是否知道森林遊樂區的調節功能？（如水源涵養、調節氣候等）□是；□否

A10. 您是否知道森林遊樂區的支持功能？（如提供物種棲息地與維護基因多樣性等）　　　　□是；□否

A11. 您是否知道森林遊樂區的文化功能？（如環境教育、生態旅遊、森林育樂等）　　　　□是；□否

| 依據**「森林遊樂區設置管理辦法」**規定，所謂**「森林遊樂區」**，指在森林區域內，為**景觀保護、森林生態保育與提供遊客從事生態旅遊、休閒、育樂活動、環境教育及自然體驗**等，經中央主管機關（行政院農委會）核定而設置之育樂區。爰此，**森林遊樂區與一般的風景特定區、觀光地區不同**，而其設置條件如下：  一、富教育意義之重要學術、歷史、生態價值之森林環境。  二、特殊之森林、地理、地質、野生物、氣象等景觀。  三、前項森林遊樂區，以面積不少於五十公頃，具有發展潛力者為限。 |
| --- |

**第二部分 森林生態系服務功能認知**

以下為**國家森林遊樂區**所能提供的森林生態系服務功能。請依據您個人的同意程度，在下列各項中適當的方格內打勾。

| 題　　　　　目 | 非  常  不  同  意 | 不  同  意 | 沒  有  意  見 | 同  意 | 非  常  同  意 |
| --- | --- | --- | --- | --- | --- |
| B1. 能提供食物、淡水等 | □ | □ | □ | □ | □ |
| B2. 能提供林木生產 | □ | □ | □ | □ | □ |
| B3. 發揮調節流量和淨化水質的作用 | □ | □ | □ | □ | □ |
| B4. 能夠提供醫藥資源 | □ | □ | □ | □ | □ |
| B5. 能調節當地氣候和空氣品質 | □ | □ | □ | □ | □ |
| B6. 碳貯存和減少溫室氣體 | □ | □ | □ | □ | □ |
| B7. 建立緩衝區（如樹木穩定斜坡），防止天然災害發生 | □ | □ | □ | □ | □ |
| B8. 防止土壤侵蝕和維護土壤肥沃 | □ | □ | □ | □ | □ |
| B9. 擁有過濾、分解廢棄物的能力 | □ | □ | □ | □ | □ |
| B10. 提供授粉作用 | □ | □ | □ | □ | □ |
| B11. 防治病蟲害 | □ | □ | □ | □ | □ |
| B12. 提供動植物生存的必要環境 | □ | □ | □ | □ | □ |
| B13. 維護物種的多樣性 | □ | □ | □ | □ | □ |
| B14. 維護基因的多樣性 | □ | □ | □ | □ | □ |
| B15. 保持生態系統的歧異度 | □ | □ | □ | □ | □ |
| B16. 對藝術和文化具有影響力 | □ | □ | □ | □ | □ |
| B17. 提供休閒遊憩的功能 | □ | □ | □ | □ | □ |
| B18. 對學術有所貢獻 | □ | □ | □ | □ | □ |
| B19. 提供環境教育場所 | □ | □ | □ | □ | □ |
| B20. 能讓人放鬆身心 | □ | □ | □ | □ | □ |
| B21. 維繫民眾與土地之間的歸屬感 | □ | □ | □ | □ | □ |

**第三部分 經營管理課題**

以下為**國家森林遊樂區**所面臨的經營管理課題。請依據您個人的認知程度，在下列各項中適當的方格內打勾。

| 編號 | 非  常  不  重  要 | 不  重  要 | 沒  有  意  見 | 重  要 | 非  常  重  要 | 題　　　　　目 | 編號 | 非  常  不  滿  意 | 不  滿  意 | 沒  有  意  見 | 滿  意 | 非  常  滿  意 |
| --- | --- | --- | --- | --- | --- | --- | --- | --- | --- | --- | --- | --- |
| I1. | □ | □ | □ | □ | □ | 遊樂區的公共設施數量（停車場、公廁、服務中心等），您覺得 | S1. | □ | □ | □ | □ | □ |
| I2. | □ | □ | □ | □ | □ | 遊樂區的交通便利性（大眾運輸、交通道路等），您覺得 | S2. | □ | □ | □ | □ | □ |
| I3. | □ | □ | □ | □ | □ | 遊樂區的遊憩動線規劃，您覺得 | S3. | □ | □ | □ | □ | □ |
| I4. | □ | □ | □ | □ | □ | 遊樂區的解說牌誌系統（摺頁、解說牌、指示牌等），您覺得 | S4. | □ | □ | □ | □ | □ |
| I5. | □ | □ | □ | □ | □ | 遊樂區的飲食便利性，您覺得 | S5. | □ | □ | □ | □ | □ |
| I6. | □ | □ | □ | □ | □ | 遊樂區的住宿便利性，您覺得 | S6. | □ | □ | □ | □ | □ |
| I7. | □ | □ | □ | □ | □ | 遊樂區的人員服務態度，您覺得 | S7. | □ | □ | □ | □ | □ |
| I8. | □ | □ | □ | □ | □ | 遊樂區的服務人員數量，您覺得 | S8. | □ | □ | □ | □ | □ |
| I9. | □ | □ | □ | □ | □ | 遊樂區的對外宣傳行銷，您覺得 | S9. | □ | □ | □ | □ | □ |
| I10. | □ | □ | □ | □ | □ | 在地居民的參與，您覺得 | S10. | □ | □ | □ | □ | □ |
| I11. | □ | □ | □ | □ | □ | 遊樂區的遊客人數管控，您覺得 | S11. | □ | □ | □ | □ | □ |
| I12. | □ | □ | □ | □ | □ | 遊樂區的噪音管制，您覺得 | S12. | □ | □ | □ | □ | □ |
| I13. | □ | □ | □ | □ | □ | 遊樂區的廢棄物（垃圾）處理，您覺得 | S13. | □ | □ | □ | □ | □ |
| I14. | □ | □ | □ | □ | □ | 遊樂區的水質水源保護，您覺得 | S14. | □ | □ | □ | □ | □ |
| I15. | □ | □ | □ | □ | □ | 遊樂區的空氣品質狀況，您覺得 | S15. | □ | □ | □ | □ | □ |
| I16. | □ | □ | □ | □ | □ | 遊樂區的建築設施搭配自然景觀的適宜性，您覺得 | S16. | □ | □ | □ | □ | □ |
| I17. | □ | □ | □ | □ | □ | 遊樂區發展觀光的同時，顧慮在地文化的永續發展，您覺得 | S17. | □ | □ | □ | □ | □ |
| I18. | □ | □ | □ | □ | □ | 遊樂區發展觀光的同時，顧慮自然生態的保育工作，您覺得 | S18. | □ | □ | □ | □ | □ |
| I19. | □ | □ | □ | □ | □ | 遊樂區遊憩承載量的評估，您覺得 | S19. | □ | □ | □ | □ | □ |
| I20. | □ | □ | □ | □ | □ | 遊樂區整體環境的監測，您覺得 | S20. | □ | □ | □ | □ | □ |
| I21. | □ | □ | □ | □ | □ | 遊樂區體驗活動的規劃（原野體驗、等），您覺得 | S21. | □ | □ | □ | □ | □ |
| I22. | □ | □ | □ | □ | □ | 遊樂區環境教育的倡導，您覺得 | S22. | □ | □ | □ | □ | □ |
| I23. | □ | □ | □ | □ | □ | 遊樂區解說服務的安排，您覺得 | S23. | □ | □ | □ | □ | □ |
| I24. | □ | □ | □ | □ | □ | 遊樂區與鄰近景點的遊憩串聯，您覺得 | S24. | □ | □ | □ | □ | □ |
| I25. | □ | □ | □ | □ | □ | 遊樂區的商品販售（書籍、紀念品），您覺得 | S25. | □ | □ | □ | □ | □ |
| I26. | □ | □ | □ | □ | □ | 參與遊樂區旅遊活動，對於改善人際關係，您覺得 | S26. | □ | □ | □ | □ | □ |
| I27. | □ | □ | □ | □ | □ | 參與遊樂區旅遊活動，對於促進家庭和諧，您覺得 | S27. | □ | □ | □ | □ | □ |
| I28. | □ | □ | □ | □ | □ | 參與遊樂區旅遊活動，對於個人壓力紓解，您覺得 | S28. | □ | □ | □ | □ | □ |
| I29. | □ | □ | □ | □ | □ | 參與遊樂區旅遊活動，對於文化保存的體悟，您覺得 | S29. | □ | □ | □ | □ | □ |
| I30. | □ | □ | □ | □ | □ | 參與遊樂區旅遊活動，對於自然生態的認知，您覺得 | S30. | □ | □ | □ | □ | □ |

**【個人基本資料】**

D1. 請問您的性別？ □男 □女

D2. 請問您的年齡？

□20歲以下 □21～30歲 □31～40歲 □41～50歲

□51～60歲 □61歲以上

D3. 請問您的教育程度？

□國小 □國中 □高中（職） □專科 □大學 □研究所以上

D4. 請問您的婚姻狀況？

□未婚 □已婚，無小孩 □已婚，有小孩 □其他

D5. 請問您目前從事的職業為(請勾選最適合的一項)？

□學生 □軍公教人員 □農林漁牧礦業 □商業 □工業 □服務業

□自由業 □退休人員 □其他

D6. 請問您個人目前的每個月平均收入為？（新台幣）

□0-20,000元 □20,001-30,000元 □30,001-40,000元 □40,001-50,000元

□50,001-60,000元 □60,001-70,000元 □70,001-80,000元

□80,001-90,000元 □90,001-100,000元 □100,000元以上

D7. 請問您目前每個月平均工作天數為？

□10天以內 □10-15天 □16-20天 □21-25天 □26天以上

D8. 請問您目前居住的地區是？ 縣（市） 鄉（鎮、市、區）

**備註：再度感謝您耐心填答此問卷!為使本問卷為有效樣本，請再瀏覽一下是否有遺漏沒填的項目。辛苦您了!**
