# Supplementary material for: Perceptions of tourists of the resources, ecological service functions and recreation value of the Guanwu National Forest Recreation Area
Source: PLoS One. 2021 Sep 30;16(9):e0257835. doi: 10.1371/journal.pone.0257835 (PMC8483363; doi:10.1371/journal.pone.0257835)
Supplement: S2 File — (DOCX) [file pone.0257835.s002.docx]

Hello, dear respondents.

First of all, thank you very much for taking time out of your busy schedule to fill out this questionnaire. This is a research project commissioned by the Forestry Bureau and conducted by the Department of Forestry of National Pingtung University of Science and Technology. The main purpose of this questionnaire is to understand your opinion on the functional value and development direction of the Guanwu National Forest Recreation Area, so that it can be used as a reference for the management unit to formulate and revise relevant policies in the future.

This questionnaire does not disclose personal information, so please feel free to complete it. The results of the survey will be used for academic research and will also be provided to relevant government agencies as a reference for the development of management strategies for forest recreation areas, so your opinion is very valuable.

Finally, I would like to thank you for your cooperation in answering the questions, as your enthusiastic participation will be the best foundation for the government's administration. Thank you!

Here's wishing you

Peaceful

From Jan-Chang Chen, Assistant Professor and Research Team, Department of Forestry, National Pingtung University of Science and Technology

**First part Recreation behaviors and Resource evaluation**

A1. Travel partners？

□Oneself □Family members □Fellow students □Friends □Colleagues □Group tour □Others

A2. Means of transportation？

□Locomotive □Personal-use vehicle □Tour bus □Rental car, locomotive □Others

A3. Traffic time？

□1h □2h □3h □4h □5h □Others

A4. Residence time？

□Within 2 h □2 h–4 h □4 h–6 h (half day) □6 h–8 h

□Above 8 h (one day) □Two days and one night □Three days and two nights □Others

A5. Natural recreation resource evaluation？

□Very low value □Low value □Normal value □High value □Very high value

A6. Cultural recreation resource evaluation？

□Very low value □Low value □Normal value □High value □Very high value

A7. Willingness to revisit the area？

□Very low □Low □Normal □High □Very high

A8. Know the supply function？(e.g. food, drinking water, forestry production, etc.)□Yes；□No

A9. Know the regulation function？(such as water conservation, climate regulation, etc.)□Yes；□No

A10. Know the support function？(e.g., providing species habitat and maintaining genetic diversity) □Yes；□No

A11. Know the culture function？(e.g. environmental education, eco-tourism, forestry recreation, etc.)□Yes；□No

| According to the "Regulations on the Establishment of Forest Recreation Areas", a "forest recreation area" refers to a recreation area within a forest area for the purpose of landscape protection, forest ecological conservation, and providing tourists with ecological tourism, leisure, recreational activities, environmental education and nature experience, etc., and is approved by the competent central government agency (Council of Agriculture, Executive Yuan). In this regard, forest recreation areas are different from general scenic areas and tourist areas, and the conditions for their establishment are as follows.  1. Forest environment with important academic, historical, and ecological values that are rich in educational significance.  2. Special forest, geography, geology, wildlife, and meteorology landscapes.  3. The forest recreation area mentioned above shall be limited to an area of at least 50 hectares and has development potential. |
| --- |

**Part II** Perception of forest ecosystem service functions

The following are the functions of the forest ecosystem services that the National Forest Recreation Area can provide. Please check the appropriate box for each of the following, depending on your personal level of agreement.

| Title | Strongly disagree | Disagree | No opinion | Agree | Strongly Agree |
| --- | --- | --- | --- | --- | --- |
| B1. Able to provide food, fresh water, etc. | □ | □ | □ | □ | □ |
| B2. Able to provide forest production | □ | □ | □ | □ | □ |
| B3. Play the role in regulating flow and purifying water quality | □ | □ | □ | □ | □ |
| B4. Able to provide medical resources | □ | □ | □ | □ | □ |
| B5. Able to regulate the local climate and air quality | □ | □ | □ | □ | □ |
| B6. Carbon storage and greenhouse gas reduction | □ | □ | □ | □ | □ |
| B7. Establishes a buffer zone (such as the slope stabilizing function of trees) to prevent natural disasters | □ | □ | □ | □ | □ |
| B8. Prevent soil erosion and maintain soil fertility | □ | □ | □ | □ | □ |
| B9. Able to filter and decompose waste | □ | □ | □ | □ | □ |
| B10. Provide pollination | □ | □ | □ | □ | □ |
| B11. Pest control | □ | □ | □ | □ | □ |
| B12. Provide necessary environment for animals and plants to survive | □ | □ | □ | □ | □ |
| B13. Maintain species diversity | □ | □ | □ | □ | □ |
| B14. Maintain genetic diversity | □ | □ | □ | □ | □ |
| B15. Maintain the diversity of ecosystems | □ | □ | □ | □ | □ |
| B16. Influence on art and culture | □ | □ | □ | □ | □ |
| B17. Provide a leisure function | □ | □ | □ | □ | □ |
| B18. Contribute to the academic field | □ | □ | □ | □ | □ |
| B19. Provide spaces for environmental education | □ | □ | □ | □ | □ |
| B20. Provide a relaxing environment | □ | □ | □ | □ | □ |
| B21. Maintaining a sense of belonging between people and the natural environment | □ | □ | □ | □ | □ |

**Part III Operating Management Topics**

The following are the management issues facing the National Forest Recreation Area. Please check the appropriate box for each of the following issues according to your personal knowledge.

| No. | Not very important | Not important | No comment | Important | Very important | Title | No. | Strongly disagree | Dissatisfied | No comment | Satisfied | Strongly satisfied |
| --- | --- | --- | --- | --- | --- | --- | --- | --- | --- | --- | --- | --- |
| I1. | □ | □ | □ | □ | □ | The number of public facilities (parking lots, public toilets, service centers, etc.) in the recreation area, in your opinion | S1. | □ | □ | □ | □ | □ |
| I2. | □ | □ | □ | □ | □ | The accessibility of the recreation area (public transportation, traffic roads, etc.), in your opinion | S2. | □ | □ | □ | □ | □ |
| I3. | □ | □ | □ | □ | □ | The recreation area's recreation route planning, do you think | S3. | □ | □ | □ | □ | □ |
| I4. | □ | □ | □ | □ | □ | The signage system of the recreation area (folders, explanatory signs, signage, etc.), do you think | S4. | □ | □ | □ | □ | □ |
| I5. | □ | □ | □ | □ | □ | The convenience of food and drink in the recreation area, do you think | S5. | □ | □ | □ | □ | □ |
| I6. | □ | □ | □ | □ | □ | The convenience of accommodation in the recreation area, do you think | S6. | □ | □ | □ | □ | □ |
| I7. | □ | □ | □ | □ | □ | The service attitude of the staff in the recreation area, do you think | S7. | □ | □ | □ | □ | □ |
| I8. | □ | □ | □ | □ | □ | The number of service personnel in the recreation area, do you think | S8. | □ | □ | □ | □ | □ |
| I9. | □ | □ | □ | □ | □ | The external publicity and marketing of the resort, do you think | S9. | □ | □ | □ | □ | □ |
| I10. | □ | □ | □ | □ | □ | The participation of local residents, in your opinion | S10. | □ | □ | □ | □ | □ |
| I11. | □ | □ | □ | □ | □ | The number of visitors in the playground, do you think | S11. | □ | □ | □ | □ | □ |
| I12. | □ | □ | □ | □ | □ | The noise control of the recreation area, do you think | S12. | □ | □ | □ | □ | □ |
| I13. | □ | □ | □ | □ | □ | Waste (garbage) disposal in the recreation area, do you think? | S13. | □ | □ | □ | □ | □ |
| I14. | □ | □ | □ | □ | □ | The protection of water quality in the recreation area, do you think | S14. | □ | □ | □ | □ | □ |
| I15. | □ | □ | □ | □ | □ | The air quality of the recreation area, do you think | S15. | □ | □ | □ | □ | □ |
| I16. | □ | □ | □ | □ | □ | The suitability of the recreation area's buildings and facilities to the natural landscape, in your opinion | S16. | □ | □ | □ | □ | □ |
| I17. | □ | □ | □ | □ | □ | The sustainable development of the local culture while developing tourism in the recreation area. | S17. | □ | □ | □ | □ | □ |
| I18. | □ | □ | □ | □ | □ | The development of tourism in recreation areas while taking into account the conservation of natural ecology, do you think | S18. | □ | □ | □ | □ | □ |
| I19. | □ | □ | □ | □ | □ | The assessment of the recreational area's recreational capacity, do you think | S19. | □ | □ | □ | □ | □ |
| I20. | □ | □ | □ | □ | □ | The monitoring of the overall environment of the recreation area, do you think | S20. | □ | □ | □ | □ | □ |
| I21. | □ | □ | □ | □ | □ | The planning of experiential activities (wilderness experience, etc.) in recreation areas, do you think | S21. | □ | □ | □ | □ | □ |
| I22. | □ | □ | □ | □ | □ | The promotion of environmental education in recreation areas, do you think | S22. | □ | □ | □ | □ | □ |
| I23. | □ | □ | □ | □ | □ | The arrangement of interpretation services in the recreation area, in your opinion | S23. | □ | □ | □ | □ | □ |
| I24. | □ | □ | □ | □ | □ | The linkage of the recreation area with the neighboring scenic spots. | S24. | □ | □ | □ | □ | □ |
| I25. | □ | □ | □ | □ | □ | The sale of goods (books, souvenirs) in the recreation area, do you think | S25. | □ | □ | □ | □ | □ |
| I26. | □ | □ | □ | □ | □ | How do you feel about the improvement of interpersonal relationship by participating in tourism activities in the recreation area? | S26. | □ | □ | □ | □ | □ |
| I27. | □ | □ | □ | □ | □ | Do you think that participating in tourism activities in recreation areas can promote family harmony? | S27. | □ | □ | □ | □ | □ |
| I28. | □ | □ | □ | □ | □ | How do you feel about personal stress relief from participating in recreation area tourism activities? | S28. | □ | □ | □ | □ | □ |
| I29. | □ | □ | □ | □ | □ | Do you think you can appreciate cultural preservation by participating in tourism activities in recreation areas? | S29. | □ | □ | □ | □ | □ |
| I30. | □ | □ | □ | □ | □ | Your perception of nature and ecology when you participate in tourism activities in recreation areas | S30. | □ | □ | □ | □ | □ |

**【Background attribute】**

D1. Gender？ □Male □Female

D2. Age？

□Under 20 □21～30 □31～40 □41～50

□51～60 □ Above61

D3. Educational background？

□Elementary school □Junior high school □Senior high school (Vocational high school) □Junior college □College □Above graduate school

D4. Marital status？

□Unmarried □Married, no children □Married, with children □Others

D5. Occupation？

□Students □Soldiers, civil servants, teachers □Agriculture, forestry, fishery, animal husbandry and mining □Commerce □Industry □Service industry

□Flexible job □Retirees □Others

D6. Monthly income (New Taiwan Dollar, yuan)?

□Below 20,000 □20,001-30,000 □30,001-40,000 □40,001-50,000

□50,001-60,000 □60,001-70,000 □70,001-80,000

□80,001-90,000 □90,001-100,000 □Above 100,000

D7. Monthly working days?

□Below 10 days □10-15 days □16-20 days □21-25 days □Above 26 days

D8. Residence？

□North □Middle □South □East □Offshore island □ oreign

**Note: Thank you again for your patience in completing this questionnaire! In order to make this questionnaire a valid sample, please go through it again to see if there are any missing items.** **Thank you very much!**
