# Supplementary material for: Perceptions of tourists of the resources, ecological service functions and recreation value of the Guanwu National Forest Recreation Area
Source: PLoS One. 2021 Sep 30;16(9):e0257835. doi: 10.1371/journal.pone.0257835 (PMC8483363; doi:10.1371/journal.pone.0257835)
Supplement: Attachment — Submitted filename: Response to Reviewers.DOCX [file pone.0257835.s005.DOCX]

Response to Reviewers

Reviewers' comments:

Reviewer's Responses to Questions

**Comments to the Author**

1. Is the manuscript technically sound, and do the data support the conclusions?

The manuscript must describe a technically sound piece of scientific research with data that supports the conclusions. Experiments must have been conducted rigorously, with appropriate controls, replication, and sample sizes. The conclusions must be drawn appropriately based on the data presented.

Reviewer #1: No

Reviewer #2: Yes

2. Has the statistical analysis been performed appropriately and rigorously?

Reviewer #1: No

Reviewer #2: Yes

3. Have the authors made all data underlying the findings in their manuscript fully available?

The [PLOS Data policy](http://www.plosone.org/static/policies.action#sharing) requires authors to make all data underlying the findings described in their manuscript fully available without restriction, with rare exception (please refer to the Data Availability Statement in the manuscript PDF file). The data should be provided as part of the manuscript or its supporting information, or deposited to a public repository. For example, in addition to summary statistics, the data points behind means, medians and variance measures should be available. If there are restrictions on publicly sharing data—e.g. participant privacy or use of data from a third party—those must be specified.

Reviewer #1: Yes

Reviewer #2: Yes

4. Is the manuscript presented in an intelligible fashion and written in standard English?

PLOS ONE does not copyedit accepted manuscripts, so the language in submitted articles must be clear, correct, and unambiguous. Any typographical or grammatical errors should be corrected at revision, so please note any specific errors here.

Reviewer #1: Yes

Reviewer #2: Yes

5. Review Comments to the Author

Please use the space provided to explain your answers to the questions above. You may also include additional comments for the author, including concerns about dual publication, research ethics, or publication ethics. (Please upload your review as an attachment if it exceeds 20,000 characters)

Reviewer #1: This study sought to examine the perceptions of tourists towards their use of a national forest recreation area (NFRA) in Taiwan. The authors implemented a questionnaire of 500 tourists and analyzed their responses using t-tests, ANOVA and importance-performance analysis (IPA). There was a nice summary of the literature, but the way it was presented read more like a list of information than a narrative. Though the research is interesting, and I see potential in this paper, I have several concerns about it that lead me to not accept it for publication. I do want to commend the authors for undertaking this work without any specific funding support, as I understand how challenging that can be.

Particular areas that need improvement include: further explanation of the methods, more technical reporting of results, and higher level interpretation of the implications of the results in the discussion. It also might be my personal preference, but I think the results should be separate from the discussion as it was difficult to distinguish between presentation and interpretation of results. I have gone into further detail about my specific recommendations below.

**Respones:**

**We thank the reviewers for their valuable comments on this paper. The literature review and analysis methods have been supplemented according to the comments of the members. In addition, the results and the discussion have been written separately in this paper, and the presentation and explanation have been strengthened.**

Methods:
I recommend including the entire survey questionnaire as supplemental information so as to assist with the replicability of the study and so readers can understand the variety of questions asked. Another recommendation I have is to provide more information about how the survey respondents were selected using convenience sampling, and include a citation to a reputable source that you used as a guide for the sampling (line 182). Please also include more information about what software(s) you used to generate the figures and conduct the statistical analyses. This will also aid in replicability. I am also curious about the response rates of the survey implementation, and what the authors did to reduce any bias in their sampling regime. About how many tourists visit the park each day and what proportion of that number was sampled? Did this study undergo any ethics review and how were the respondents given informed consent? There’s a lot missing here. I recommend reading more literature about quantitative survey design and presentation in natural resource management for guidance.

**Respones:**

**1. Due to the length of the paper, the content of the survey questionnaire is supplemented in the "Research Results" section.**

**2. As suggested by the reviewers, we supplemented the literature on the lobbying data and sampling methods.**

**3. As suggested by the reviewers, the description of data analysis methods is enhanced.**

Results:
The paragraph beginning on line 251 seems like it would be better suited in the Methods as it describes evaluation of the survey questionnaire and analysis of the data. Lines 294-298 also should go in the Methods.

I recommend improved presentation of survey results so it’s easier to visualize trends. Table 1 does a nice job of demonstrating the demographic snapshot of respondents, and these types of data are usually depicted in a table. However, I think the data presented in Tables 2 and 3 would be better viewed in a figure rather than a table. R Studio has a great Likert package to assist with visualizing Likert-style data, but I think you can also make stacked bar plots in Excel in a similar fashion, overlaying the percent of respondents who gave each answer. The data presented in Table 4 should also include the degrees of freedom (df) used in the analyses. Another suggestion for analyzing responses could be a Chi-square analysis. Also, where are the data stored? Please include that information in the manuscript. Some public (and free, I think) places are GitHub and Data Dryad.

**Respones:**

1. **Some of the results were adjusted to the Research methods section according to the comments of the members. 2.**
2. **Some of the table contents are presented in graphical form.**
3. **Results and discussion are written separately.**

Discussion:
Overall, the discussion of the implications of the results and applicability to the literature read more like a list of previous works rather than fitting the main conclusions of this study within the literature. One major suggestion I have is to break up the discussion into its own section and to make sure each paragraph has its own point to make. Each point from the paragraph should apply to a key finding of the study and how it fits in with the literature. I also recommend ending the paper with a very broad conclusion, not the limitations of the study.
There is interesting information in here, but I think the paper falls short of presenting it in the best way to be understood. Depending on the responses of the other reviewers, I would be willing to read further versions of this manuscript.

**Respones:**

**We thank the reviewers for their valuable comments, and this paper has been supplemented with discussion based on their comments, and the conclusions of the study have been rewritten.**
