# Supplementary material for: Perceptions of tourists of the resources, ecological service functions and recreation value of the Guanwu National Forest Recreation Area
Source: PLoS One. 2021 Sep 30;16(9):e0257835. doi: 10.1371/journal.pone.0257835 (PMC8483363; doi:10.1371/journal.pone.0257835)
Supplement: Attachment — Submitted filename: Response-to-Reviewers20210818.docx [file pone.0257835.s006.docx]

Response to Reviewers

PONE-D-21-12141R1
Perceptions of tourists of the resources , ecological service functions and recreation value of the Guanwu National Forest Recreation Area
PLOS ONE

Dear Dr. Chen,

Thank you for submitting your manuscript to PLOS ONE. After careful consideration, we feel that it has merit but does not fully meet PLOS ONE’s publication criteria as it currently stands. Therefore, we invite you to submit a revised version of the manuscript that addresses the points raised during the review process.

Please submit your revised manuscript by 6 weeks. If you will need more time than this to complete your revisions, please reply to this message or contact the journal office at [plosone@plos.org](mailto:plosone@plos.org). When you're ready to submit your revision, log on to <https://www.editorialmanager.com/pone/> and select the 'Submissions Needing Revision' folder to locate your manuscript file.

Please include the following items when submitting your revised manuscript:

- A rebuttal letter that responds to each point raised by the academic editor and reviewer(s). You should upload this letter as a separate file labeled 'Response to Reviewers'.
- A marked-up copy of your manuscript that highlights changes made to the original version. You should upload this as a separate file labeled 'Revised Manuscript with Track Changes'.
- An unmarked version of your revised paper without tracked changes. You should upload this as a separate file labeled 'Manuscript'.

If you would like to make changes to your financial disclosure, please include your updated statement in your cover letter. Guidelines for resubmitting your figure files are available below the reviewer comments at the end of this letter.

If applicable, we recommend that you deposit your laboratory protocols in [protocols.io](http://protocols.io/) to enhance the reproducibility of your results. Protocols.io assigns your protocol its own identifier (DOI) so that it can be cited independently in the future. For instructions see: <http://journals.plos.org/plosone/s/submission-guidelines#loc-laboratory-protocols>. Additionally, PLOS ONE offers an option for publishing peer-reviewed Lab Protocol articles, which describe protocols hosted on [protocols.io](http://protocols.io/). Read more information on sharing protocols at <https://plos.org/protocols?utm_medium=editorial-email&utm_source=authorletters&utm_campaign=protocols>.

We look forward to receiving your revised manuscript.

Kind regards,

Shah Md Atiqul Haq
Academic Editor
PLOS ONE

Journal Requirements:

Please review your reference list to ensure that it is complete and correct. If you have cited papers that have been retracted, please include the rationale for doing so in the manuscript text, or remove these references and replace them with relevant current references. Any changes to the reference list should be mentioned in the rebuttal letter that accompanies your revised manuscript. If you need to cite a retracted article, indicate the article’s retracted status in the References list and also include a citation and full reference for the retraction notice.

Additional Editor Comments:

Dear authors,
I would ask you to revise the paper by following the reviewers' comments and suggestions.
Best wishes


[Note: HTML markup is below. Please do not edit.]

Reviewers' comments:

Reviewer's Responses to Questions

**Comments to the Author**

1. If the authors have adequately addressed your comments raised in a previous round of review and you feel that this manuscript is now acceptable for publication, you may indicate that here to bypass the “Comments to the Author” section, enter your conflict of interest statement in the “Confidential to Editor” section, and submit your "Accept" recommendation.

Reviewer #1: (No Response)

2. Is the manuscript technically sound, and do the data support the conclusions?

The manuscript must describe a technically sound piece of scientific research with data that supports the conclusions. Experiments must have been conducted rigorously, with appropriate controls, replication, and sample sizes. The conclusions must be drawn appropriately based on the data presented.

Reviewer #1: Yes

3. Has the statistical analysis been performed appropriately and rigorously?

Reviewer #1: Yes

4. Have the authors made all data underlying the findings in their manuscript fully available?

The [PLOS Data policy](http://www.plosone.org/static/policies.action#sharing) requires authors to make all data underlying the findings described in their manuscript fully available without restriction, with rare exception (please refer to the Data Availability Statement in the manuscript PDF file). The data should be provided as part of the manuscript or its supporting information, or deposited to a public repository. For example, in addition to summary statistics, the data points behind means, medians and variance measures should be available. If there are restrictions on publicly sharing data—e.g. participant privacy or use of data from a third party—those must be specified.

Reviewer #1: Yes

5. Is the manuscript presented in an intelligible fashion and written in standard English?

PLOS ONE does not copyedit accepted manuscripts, so the language in submitted articles must be clear, correct, and unambiguous. Any typographical or grammatical errors should be corrected at revision, so please note any specific errors here.

Reviewer #1: Yes

6. Review Comments to the Author

Please use the space provided to explain your answers to the questions above. You may also include additional comments for the author, including concerns about dual publication, research ethics, or publication ethics. (Please upload your review as an attachment if it exceeds 20,000 characters)

Reviewer #1: I want to thank the authors for their substantial edits to their manuscript, which I think strengthened the study overall. I admire their revision of the Results section, especially the detail devoted to making new figures depicting the results, and the addition of a Discussion section. Below, I outline several additional recommendations to continue to strengthen the manuscript:
1. Reference the supplemental information (like the survey questionnaire, the data, etc.) when it is mentioned in the manuscript. For example, you mention the survey questionnaire at lines 169-173, but do not reference where in the supplemental information the survey is. Please do this so the reader knows where to look.

**Respones:**

**The location of the supplemental information has been added, see line 171-172.**

2. Please explain the number of respondents you tried to interview, and the response rate of the interviews in the paragraph that begins at line 184. I know you added more references to support convenience sampling, and I appreciate that you mentioned the number of total questionnaires completed. However, an important missing piece is reporting the total number of interviews you tried to do, so we know the response rate (i.e. if you tried talking to 1000 people, but only had 500 questionnaires completed, that would be a 50% response rate). Reporting on this assists with replicability if other researchers want to do a similar study in their national park(s).

**Respones:**

**According to the statistics of interviewers, the response rate for this study was 55%, see line 192-193.**

3. Please include which software you used for data processing and analysis (beginning at line 198).

**Respones:**

**We have added the adopted software, see line 213-215.**

4. Please include explanation of all panels in all figure descriptions. For example, Figure 1 has 10 panels and is separated into two sections (“Recreation behaviors” and “Resource evaluation”), so please describe the different panels in more detail in the figure descriptions. Figure 3 especially requires more explanation in the description as it appears to be a multidimensional plot; please describe what was displayed in the plot and what the axes represent. I have no idea how to interpret this plot otherwise.

**Respones:**

**The illustrations in Figure 1 and Figure 3 have been corrected, see line 277-283 and line 350-353.**

5. I think the Discussion and Conclusions should be combined, with the details in the Conclusion included first as they summarize the key points from the study. Then, you can start to apply the main findings to the broader literature and discuss any limitations from your study. How the Discussion and Conclusions are depicted right now makes it hard to understand the key findings of the study, as they are presented after connecting it to the literature. Please rewrite. If you want to keep a Conclusions section, stick to one paragraph and summarize everything there. But only after you summarize the key findings in more detail earlier in the paper.
I see the value in getting this paper published, but there are still some limitations to me accepting it as is. I am willing to review the paper one more time if the above points are addressed. Thank you.

**Respones:**

**We still keep a Conclusions section, and strengthen the content of the Discussion section, and strengthen the linkage between Discussion and Conclusions.**

7. PLOS authors have the option to publish the peer review history of their article ([what does this mean?](https://journals.plos.org/plosone/s/editorial-and-peer-review-process#loc-peer-review-history)). If published, this will include your full peer review and any attached files.


If you choose “no”, your identity will remain anonymous but your review may still be made public.


**Do you want your identity to be public for this peer review?** For information about this choice, including consent withdrawal, please see our [Privacy Policy](https://www.plos.org/privacy-policy).

Reviewer #1: No

[NOTE: If reviewer comments were submitted as an attachment file, they will be attached to this email and accessible via the submission site. Please log into your account, locate the manuscript record, and check for the action link "View Attachments". If this link does not appear, there are no attachment files.]

While revising your submission, please upload your figure files to the Preflight Analysis and Conversion Engine (PACE) digital diagnostic tool, <https://pacev2.apexcovantage.com/>. PACE helps ensure that figures meet PLOS requirements. To use PACE, you must first register as a user. Registration is free. Then, login and navigate to the UPLOAD tab, where you will find detailed instructions on how to use the tool. If you encounter any issues or have any questions when using PACE, please email PLOS at [figures@plos.org](mailto:figures@plos.org). Please note that Supporting Information files do not need this step.
